# A phase Ib, open label, dose escalation trial of the anti-CD37 monoclonal antibody, BI 836826, in combination with ibrutinib in patients with relapsed/refractory chronic lymphocytic leukemia

**DOI:** 10.1007/s10637-020-01056-4

**Published:** 2021-03-08

**Authors:** Alexey V. Danilov, Stephen E. Spurgeon, Tanya Siddiqi, Anne-Marie Quinson, Daniela Maier, Dionne Smith, Jennifer R. Brown

**Affiliations:** 1 City Of Hope National Medical Center, Duarte, CA USA; 2 Knight Cancer Institute at Oregon Health & Science University, Portland, OR USA; 3 Boehringer Ingelheim Pharma GmbH & Co. KG, Biberach an der Riss, Germany; 4 Boehringer Ingelheim Pharmaceuticals Inc, Ridgefield, CT USA; 5 Dana-Farber Cancer Institute, Boston, MA USA

**Keywords:** BI 836826, CD37, Chronic lymphocytic leukemia, Phase Ib, Relapsed

## Abstract

BI 836826 is a chimeric immunoglobulin G1 antibody targeting CD37, a transmembrane protein expressed on normal and malignant B cells. This open-label, phase Ib, dose-escalation study was conducted to determine the recommended phase II dose (RP2D) of BI 836826 + ibrutinib in patients with relapsed/refractory chronic lymphocytic leukemia (CLL). Eligible patients received 420 mg/day of ibrutinib with escalating doses of BI 836826. BI 836826 was administered in 4-week cycles. After Cycle 12, patients achieving complete response (CR), CR with incomplete marrow recovery, or minimal residual disease-negative partial response could continue to receive BI 836826 + ibrutinib every 4 weeks for ≤ 12 additional cycles. Patients received either 100 mg (*n* = 3) or 200 mg (*n* = 3) BI 836826 + ibrutinib. In the 100 mg BI 836826 cohort, one patient received two cycles and two patients received 22 cycles of BI 836826. In the 200 mg BI 836826 cohort, patients received 12, 16 and 20 cycles of BI 836826, respectively. All patients discontinued BI 836826 and continued ibrutinib outside the trial. No dose-limiting toxicities were reported in the maximum tolerated dose (MTD) evaluation period. As the trial was discontinued before the MTD was reached, the RP2D was not determined. Grade 3/4 adverse events (AEs) were predominantly hematological. Pseudomonal bacteremia was the only drug-related AE of special interest. BI 836826 + ibrutinib did not exceed the MTD at doses up to 200 mg in patients with CLL. However, RP2D and MTD were not formally established, as the sponsor discontinued the trial.

## Introduction

The Bruton tyrosine kinase (BTK) inhibitor, ibrutinib, is well established in the treatment of patients with previously untreated and relapsed/refractory chronic lymphocytic leukemia (CLL) [1–3]. While ibrutinib monotherapy is associated with impressive response rates in CLL patients, including those with del(17p) [1, 4], deep responses are rare, thus necessitating continuous use. This can result in cumulative toxicities, leading to treatment discontinuation [5]. Furthermore, patients often become resistant to long-term treatment, potentially leading to disease progression or transformation [6]. Therefore, there is clinical rationale for combining ibrutinib with other agents that could increase depth of response and delay development of resistance.

Preclinical and clinical studies have demonstrated that CD37, a tetraspanin B cell surface molecule, is a potential drug target in patients with CLL [7–10].

BI 836826 is a chimeric mouse–human anti-CD37 monoclonal antibody engineered to enhance binding and effector function that mediates direct antibody-dependent cell-mediated cytotoxicity (ADCC) against CLL cells [11]. A phase I, dose escalation study of BI 836826 in patients with relapsed/refractory CLL demonstrated acceptable tolerability and notable efficacy, particularly in patients with poor-risk features such as del(17p) and *TP53* mutations [12]. In this phase Ib, dose-escalation study, we investigated the combination of ibrutinib and BI 836826 in patients with relapsed/refractory CLL.

## Patients and methods

### Patients

Eligible patients were ≤ 18 years old with relapsed/refractory CLL according to the International Workshop on Chronic Lymphocytic Leukemia (IWCLL) criteria [13]. All patients had received at least one prior line of systemic treatment. Prior BTK inhibitors were not allowed. Other eligibility criteria included Eastern Cooperative Oncology Group (ECOG) Performance Status (PS) of 0–2; clinically quantifiable disease burden (absolute lymphocyte count > 10,000/µL, measurable lymphadenopathy, or quantifiable bone marrow infiltration); adequate organ function; and residual non-hematological toxicity from prior treatment of grade ≤ 1.

Key exclusion criteria were: any CD37-targeting antibody or CD37 antibody-drug conjugate; allogeneic stem cell transplant within 1 year or active graft-versus-host disease; known transformation of CLL to an aggressive B-cell malignancy at the time of screening; history of non-CLL malignancy except for adequately treated in-situ, stage I or II carcinoma in complete response (CR) or any other cancer that had been in CR for ≥ 2 years after the end of cancer treatment; and active uncontrolled autoimmune cytopenia.

The trial was carried out in accordance with the Declaration of Helsinki, Good Clinical Practice Guidelines, applicable regulatory requirements and Boehringer Ingelheim standard operating procedures. The study protocol was approved by the Institutional Review Boards of all participating institutions. Written informed consent was obtained from all patients.

### Study design and treatment

The primary objectives of this single arm, open-label, dose escalation phase Ib study were to determine the recommended phase II dose (RP2D) of BI 836826 plus ibrutinib in patients with relapsed/refractory CLL, and the number of patients with dose-limiting toxicities (DLTs) during the maximum tolerated dose (MTD) evaluation period (Cycle 1). Other objectives were the determination of the MTD, safety and efficacy of the combination.

Eligible patients underwent a two-week run-in phase with ibrutinib and remained on ibrutinib throughout the trial at a constant dose of 420 mg daily. BI 836826 was planned at dose levels of 100, 200, 400, 600, 800 and 1400 mg. Mandatory pre-medication (antihistamine, analgesic and glucocorticoid) to mitigate the risk of infusion-related reactions (IRRs) was given 30–120 min prior to BI 836826 administration. BI 836826 was then administered via rate-controlled intravenous infusion in Cycle 1 as a 10 mg dose on Day 1, on Days 2 and 8 at 50% of the assigned dose on each day, and on Day 15 at 100% of the assigned dose. In Cycles 2–4, BI 836826 was administered at the assigned dose on Days 1 and 15 of each cycle. In Cycles 5–12, BI 836826 was administered at the assigned dose on Day 1 only. BI 836826 was administered in 4-week cycles. At the end of Cycle 12, bone marrow biopsies were performed to evaluate response. Patients with a CR, CR with incomplete marrow recovery (CRi), or minimal residual disease (MRD)-negative partial response (PR) could choose to receive 12 additional treatment cycles of the combination. Following completion of 12 or 24 cycles, or discontinuation due to disease progression, unacceptable toxicity, or withdrawal of consent, patients could continue to receive ibrutinib outside of the study at the discretion of the treating investigator.

### Study assessments

Safety was assessed by determining the incidence and severity of adverse events, graded according to the Common Terminology Criteria for Adverse Events (CTCAE), version 4.0. Assessment of laboratory parameters, vital signs, physical examination and ECG was also undertaken. DLTs were defined as: any non-hematologic adverse event of grade ≥ 3 related to BI 836826 or ibrutinib, except IRRs and grade 3 alanine aminotransferase and/or aspartate aminotransferase elevation without concomitant bilirubin elevation or any other asymptomatic grade 3 laboratory abnormality with spontaneous recovery within 1 week; hematologic adverse events related to BI 836826 or ibrutinib including grade 4 neutropenia with concomitant infection; grade 4 febrile neutropenia, and grade 3 febrile neutropenia not resolving within 72 hours; grade 4 thrombocytopenia with clinically significant bleeding; grade 4 anemia; any grade 5 hematologic adverse event. Adverse events of special interest (AESIs) included DLTs, infusion-related reactions of grade ≥ 3, late-onset infections, events indicative of drug-induced liver injury, and tumor lysis syndrome. Adverse events consistent with the definition of a DLT but occurring after the MTD evaluation period were also considered AESIs.

Overall response, duration of response, MRD and reduction in size of tumor lymph nodes were exploratory endpoints. Overall response was defined as patients who achieved CR, CRi, PR or PR with lymphocytosis (PR-L) according to modified IWCLL guidelines [13]. MRD was evaluated in both blood and bone marrow samples and was defined as < 1 leukemic cell per 10,000 leukocytes detected by multi-parameter flow cytometry.

### Statistical methods

All analyses were descriptive and exploratory. No formal statistical analysis was conducted. Dose escalation was guided by a Bayesian 5-parameter logistic regression model (BLRM) with overdose control [14, 15] that was fitted to binary toxicity outcomes.

## Results

### Patients and treatment exposure

Between July 7, 2016 and July 9, 2019, 10 patients were screened, of whom 7 entered the run-in period with ibrutinib. Six patients were treated with BI 836826 plus ibrutinib across three sites in the United States (100 mg BI 836826: *n* = 3; 200 mg BI 836826: *n* = 3). The median age among treated patients was 71.0 years (range 57–76 years); all patients were Caucasian (Table 1).

**Table 1:** Patient demographics

	BI 836826 dose^a^		
	100 mg (*n* = 3)	200 mg (*n* = 3)	Total (*N* = 6)
Male, *n* (%)	1 (33)	2 (67)	3 (50)
Race, *n* (%)			
White	3 (100)	3 (100)	6 (100)
Ethnicity, *n* (%)			
Not Hispanic/Latino	2 (67)	3 (100)	5 (83)
Hispanic/Latino	1 (33)	0	1 (17)
Median age, years (range)	75.0 (68–76)	67.0 (57–74)	71.0 (57–76)
ECOG PS at baseline, *n* (%)			
0	1 (33)	1 (33)	2 (33)
1	2 (67)	2 (67)	4 (67)
RAI stage at diagnosis, *n* (%)			
0 I II III IV	1 (33) 0 1 (33) 0 1 (33)	1 (33) 1 (33) 0 1 (33) 0	2 (33) 1 (17) 1 (17) 1 (17) 1 (17)
Mean time from first diagnosis, years (SD)	10.4 (4.9)	11.1 (5.1)	10.7 (4.5)
Median number of previous CLL therapies (range)	1 (1–1)	4 (1–5)	1 (1–5)
Status after prior treatment			
Relapsed Refractory	2 (67) 1 (33)	2 (67) 1 (33)	4 (67) 2 (33)

^a^Given in combination with ibrutinib 420 mg/day

*CLL* chronic lymphocytic leukemia, *ECOG PS* Eastern Cooperative Oncology Group Performance Status, *RAI* staging system for CLL [16], *SD* standard deviation

In the 100 mg BI 836826 cohort, one patient received two cycles and two patients received 22 cycles of BI 836826. The patients in the 200 mg cohort received 12, 16 and 20 cycles of BI 836826, respectively. All six patients discontinued treatment with BI 836826 (disease progression, *n* = 1; investigator discretion, *n* = 3; lack of response by cycle 12, n = 2). Mean duration of BI 836826 exposure was 440.2 days. There were no dose reductions. Two patients in the 100 mg BI 836826 cohort had at least one important protocol deviation. Both patients continued treatment with BI 836826 beyond cycle 12 without having a CR, CRi, or MRD-negative PR. One of these patients did not receive pre-medication with glucocorticoid in Cycle 3, as required per protocol.

### DLTs during the MTD evaluation period, MTD and RP2D

No DLTs were reported during the MTD evaluation period with 100 or 200 mg BI 836826. However, the trial was discontinued before patients were treated with 400 mg BI 836826, and the MTD and RP2D were therefore not determined.

### Safety

All six patients had at least one adverse event that was considered by the investigator to be related to BI 836826. The most common BI 836826-related adverse events (any grade/grade  ≥ 33) were IRR (67%/17%), neutropenia (50%/33%), anemia (33%/33%), lymphopenia (33%/33%) and fatigue (33%/0%; Table 2). All IRRs (ten in four patients) were grade ≤ 2 except for one grade 3 IRR. The majority of IRRs occurred within the first two cycles and were resolved within a day.

**Table 2:** Adverse events considered related to BI 836826 occurring in > 2 patients

	Any-grade *n* (%)	Grade 3/4 *n* (%)
Infusion-related reactions	4 (67)	1 (17)
Neutropenia	3 (50)	2 (33)
Anemia	2 (33)	2 (33)
Lymphopenia	2 (33)	2 (33)
Fatigue	2 (33)	0

Five patients had serious adverse events (considered related to study drug in 4 patients), including neutropenia, acute coronary syndrome, cellulitis, pseudomonal bacteremia and Bowen’s disease. No serious adverse event was experienced by more than one patient. There were no fatal adverse events, and no dose reductions or permanent discontinuations of BI 836826 due to adverse events. One patient had an ibrutinib dose reduction due to grade 2 neutropenia. After the MTD evaluation period, two patients in the 200 mg cohort reported grade 3 DLTs: a duodenal ulcer occurring on Day 325 and pseudomonal bacteremia (considered as related to study drug) on Day 280.

Three AESIs were recorded: the two DLTs reported after the MTD evaluation period and grade 2 basal cell carcinoma (Day 622).

Based on laboratory data, grade 4 neutropenia was reported for two patients (one patient in each dose cohort). Neither episode was associated with a concomitant infection. One patient in the 100 mg dose cohort had an episode of grade 4 thrombocytopenia, which was not associated with concomitant bleeding. Two patients (one in each dose cohort) had an episode of grade ≥ 3 decreased CD4 + T-cell count but there were no concomitant CD4+-related specific infections.

### Efficacy

Overall response rate was 83% among the 6 evaluable patients (one CR and four PRs; Table 3) The ORR in the 200 mg cohort was 100% (one CR, two PR). Of five patients who underwent peripheral blood-based MRD analysis, two were MRD-negative, one in each cohort including the patient who achieved CR. Three patients had bone marrow aspirate samples analyzed for MRD. None were MRD-negative but one patient developed MRD-negative CR a month after study completion.

**Table 3:** Best overall response in patients receiving BI 836826 plus ibrutinib

	BI 836826 dose		
Patients with response, *n* (%)	100 mg *n* = 3	200 mg *n* = 3	Total *N* = 6
Overall response	2 (67)	3 (100)	5 (83)
CR	0	1 (33)	1 (17)
CRi	0	0	0
PR	2 (67)	2 (67)	4 (67)
PR-L	0	0	0
Stable disease			
Progressive disease	1 (33)	0	1 (17)

*CR* complete response, *CRi* complete response with incomplete marrow recovery, *OR* overall response (CR + CRi + PR + PR-L), *PR* partial response; *PR-L* partial response with lymphocytosis

**Fig. 1:**
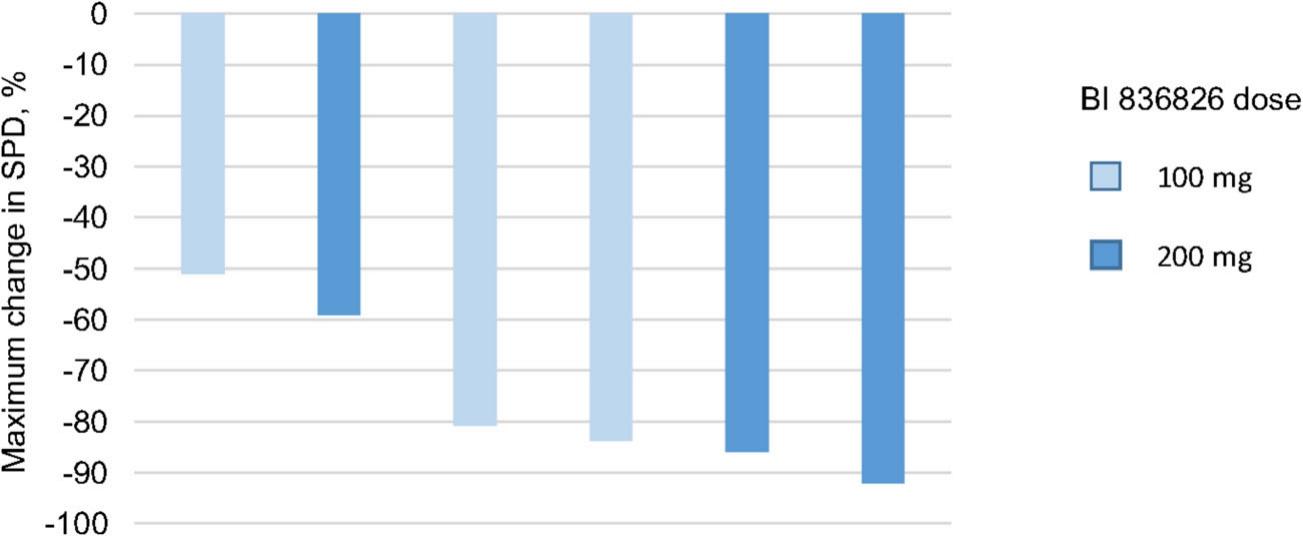
Maximum change in SPD of lymph node lesions at best response

All five patients were censored for the analysis of duration of overall response since they had discontinued before progression or death was observed. Mean (standard deviation [SD]) duration of response was 538.0 (31.1) days and 312.0 (143.96) days in the 100 mg and 200 mg BI 836826 cohorts, respectively. The median best percentage change from baseline in the SPD of lymph nodes was -81.2% (range -84% to -51%) in the 100 mg dose group and -79.2% (range -92% to -59%) in the 200 mg dose group (Fig. 1). Tumor size was reduced in all six patients.

## Discussion

In this phase Ib, dose-escalation study of BI 836826 plus ibrutinib in patients with relapsed/refractory CLL, no DLTs were observed during the MTD evaluation period in either the 100 mg or 200 mg BI 836320 cohorts. Despite these promising findings, the sponsor took the strategic decision to discontinue the clinical development of BI 836826, due to the rapidly evolving CLL treatment landscape. Accordingly, the MTD was not reached and the RP2D was not determined. There were no safety reasons to terminate the program. Nevertheless, the results from this study suggest that BI 836826 can be safely combined with ibrutinib, with no apparent additive toxicity. Adverse events associated with the combination were consistent with previous clinical experience with BI 836826 [12] or ibrutinib monotherapy [4].

The efficacy of ibrutinib in relapsed/refractory CLL is well established [4, 17, 18], with an objective response rate of 63% observed in the phase III study, RESONATE [4]. Of note, overall response rate to ibrutinib generally deepens over time [17, 19], and had increased to 91% by the final analysis of RESONATE [2, 20]. Our preliminary findings (83% overall response rate) suggest that the addition of anti-CD37 agents to ibrutinib could potentially improve efficacy. While the sample size is limited, the promising efficacy of this combination especially at the 200 mg dose (100%), supports ongoing exploration of agents, including those that target CD37, for fixed-duration therapy in CLL.

A number of CD37-based therapeutics are currently undergoing investigation in CLL, including otlertuzumab, an anti-CD37 mono-specific ADAPTIR therapeutic protein [8]. Notably, in phase II studies, the addition of otlertuzumab to bendamustine greatly enhanced response compared with bendamustine alone in relapsed CLL patients [21]. Additionally, ^212^Pb-NNV003, a CD37-targeted radioimmunotherapy, has demonstrated notable anti-tumor effects in an animal model of CLL [22].

In conclusion, BI 836826 plus ibrutinib did not exceed the MTD at doses up to 200 mg in patients with CLL, and no DLTs were reported in the MTD evaluation period. While the RPTD and MTD were not formally established, our findings suggest that an anti-CD37 antibody may be combined with ibrutinib, or potentially other BTK inhibitors, for the treatment of relapsed/refractory CLL. Since continuous therapy with BTK inhibitors leads to toxicities, therapeutic resistance and is associated with high costs, exploration of novel combinations involving active agents with different therapeutic targets remains an unmet medical need.

## Data Availability

The clinical study report (including appendices, but without line listings) and other clinical documents related to this study may be accessed on request. Prior to providing access, the documents and data will be examined, and, if necessary, redacted and de-identified to protect the personal data of study participants and personnel, and to respect the boundaries of the informed consent of the study participants. See https://trials.boehringer-ingelheim.com/data_sharing/sharing.html#accordion-1-2 for further details. Bona fide, qualified scientific and medical researchers may request access to de-identified, analyzable patient-level study data, together with documentation describing the structure and content of the datasets. Researchers should use https://clinicalstudydatarequest.com/ to request access to raw data from this study.
